# miR-27a Regulates Sheep Adipocyte Differentiation by Targeting *CPT1B* Gene

**DOI:** 10.3390/ani12010028

**Published:** 2021-12-23

**Authors:** Bo Li, Xiaoyu Huang, Chen Yang, Ting Ge, Leiyun Zhao, Xiaoqiang Zhang, Lintao Tian, Enping Zhang

**Affiliations:** College of Animal Science and Technology, Northwest A&F University, Xianyang 712100, China; lib1028@163.com (B.L.); hxy_beyond@sina.com (X.H.); aayangchenaa@163.com (C.Y.); geting@nwafu.edu.cn (T.G.); zly18893818438@163.com (L.Z.); zhangxiaoqiang1028@163.com (X.Z.); tlt2021296@126.com (L.T.)

**Keywords:** miR-27a, *CPT1B*, ovine preadipocytes, adipogenesis

## Abstract

**Simple Summary:**

The content of intramuscular fat (IMF) is the main determinant of the nutritional and economic value of sheep meat. Therefore, lipid synthesis in sheep longissimus lumborum (LL) has become an important research focus. MicroRNA-27a (miR-27a) has been shown to play a crucial role in the proliferation and differentiation of adipocyte progenitor cells. In this study, we revealed that miR-27a significantly inhibited the formation of lipid droplets by targeting *CPT1B* to inhibit genes involved in lipid synthesis including PPAR γ, SCD, LPL, and FABP4. Here, we constructed a miR-27a-*CPT1B* regulatory network map, which revealed the interaction between miR-27a and *CPT1B* in lipid synthesis in ovine preadipocytes.

**Abstract:**

MiRNAs are vital regulators and play a major role in cell differentiation, biological development, and disease occurrence. In recent years, many studies have found that miRNAs are involved in the proliferation and differentiation of adipocytes. The objective of this study was to evaluate the effect of miR-27a and its target gene *CPT1B* on ovine preadipocytes differentiation in Small-tailed Han sheep (*Ovis aries*). Down-regulation of miR-27a significantly promoted the production of lipid droplets, while overexpression of miR-27a led to a reduction in lipid droplet production. In addition, inhibition of miR-27a led to a significant increase in the expression of genes involved in lipid synthesis, including PPAR γ, SCD, LPL, and FABP4. Target Scan software predicted that *CPT1B* is a new potential target gene of miR-27a. Further experiments revealed that *CPT1B* gene expression and protein levels were negatively correlated with miR-27a expression. Overexpression of miR-27a led to a significant decrease in *CPT1B* mRNA levels and inhibited the accumulation of lipid droplets and vice versa. Moreover, overexpression of *CPT1B* promoted the synthesis of lipid droplets in ovine preadipocytes. Furthermore, luciferase reporter assays confirmed *CPT1B* to be a miR-27a direct target gene. This study confirmed that miR-27a increases the expression of genes related to lipid synthesis in ovine preadipocytes by targeting *CPT1B*, thereby promoting the synthesis of lipid droplets. The results of this study can be used to be exploited in devising novel approaches for improving the IMF content of sheep.

## 1. Introduction

The content of intramuscular fat (IMF) content is an index to measure meat quality [1], which directly affects the tenderness, juiciness, and flavor of mutton [2]. Therefore, understanding the mechanism of IMF synthesis in sheep meat has become a research hotspot. The formation of IMF in sheep is regulated by many factors, such as heredity [3], age [4], environment [5], and nutrition [6]. However, the molecular mechanism underlying IMF formation is still poorly understood.

MicroRNAs (miRNAs) are one of the most important regulatory factors [7] by repressing mRNA translation or by binding to target mRNAs [8]. In addition, it has been found that miRNAs play a key role in fat synthesis. For instance, miR-340-5p regulates lipid formation by targeting *ATF7* [9]. Moreover, miR-32-5p has been shown to promote adipocyte differentiation via inhibition of *KLF3* [10]. miR-124-3p targets *C/EBPα* and influences fat differentiation in sheep muscle tissue [11]. miR-330-5p negatively regulates adipocyte differentiation by targeting the *BCAT2* gene [12].

As a member of the miRNA-27 family, miR-27a regulates the differentiation of ovine preadipocytes and triglycerides synthesis by regulating the expression of the target gene *RXR* [13]. MiR-27a is a regulatory factor of peroxisome proliferator-activated receptor g (*PPAR*) γ [14]. It has been hypothesized that *CPT1B* may be the target gene of miR-27a. Interestingly, studies have shown that *CPT1B* affects lipid metabolism in Chinese Simmental cattle [15] and that the expression of *CPT1B* is associated with preventing fatty liver disease [16].

Therefore, in this study, the relationship between miR-27a and *CPT1B* was studied. In addition, the impact of the miR-27a-*CPT1B* interaction on the differentiation of ovine preadipocytes was evaluated. This study provides a basis for exploring the role of miR-27a in the differentiation of ovine preadipocytes.

## 2. Materials and Methods

### 2.1. Ovine Preadipocytes Isolation and Culture

Under sterile conditions, small vessels and other connective tissues of LL tissues were removed from one-month-old Small-tailed Han sheep (Yangling, Shaanxi, China). The Small-tailed Han sheep is ewe, weighing 10.8 kg. Muscle tissue was minced, digested, filtered, and centrifuged at 1200 rpm for 10 min. Harvested ovine preadipocytes were cultured as previously described [17].

Preadipocyte differentiation was initiated using an induction medium (Cyagen Biosciences, Santa Clara, CA, USA). The induction medium was composed of medium A (fetal bovine serum (FBS), penicillin/streptomycin, insulin, glutamine, 3-isobutyl 1-methylxanthine, rosiglitazone, and dexamethasone) and medium B (FBS, penicillin/streptomycin, and insulin), which were alternately used every two days until day 8 for cell culture.

### 2.2. Cloning of CPT1B

The coding sequence of *CPT1B* of Small-tailed Han sheep was amplified by PCR using KOD-Plus-Neo (Toyobo, Osaka, Japan). TRIzol reagent (Agbio, Changsha, China) was used for RNA isolation from LL of Small-tailed Han sheep, as per the manufacturer’s instructions. To synthesize cDNA, a high-capacity cDNA reverse transcription kit (Takara, Dalian, China) was used. cDNA was used as a template. *CPT1B* fragment was amplified using the following primers: forward primer 5′- ATGACAACAGTGGGTTCCTCCTG -3′ and reverse primer 5′- TTAGCCATCAGCCTTAGAAACTTG -3′. PCR products were cloned into the pcDNA3.1 vector and sequenced at ZKYTONG Biological Technology (Xi’an, China). The cloned fragment was named *pcDNA3.1-CPT1B*.

### 2.3. Cell Transfection

Ovine preadipocytes grew to 80% confluence. MiR-27a simulant, negative control (NC) simulant (miR-NC), miR-27a inhibitor, NC inhibitor (Shanghai Gene Pharmaceuticals, China), and *pcDNA3.1-CPT1B* were transfected into cells with Lipofectamine^®^ 3000 reagent (Invitrogen, Waltham, MA, USA) according to the manufacturer’s instructions. After 6 h of transfection, cell culture was continued in a fresh complete medium, which was replaced by DMEM medium containing 10% FBS and 1% penicillin/streptomycin.

### 2.4. Determination of Gene Expression

*ꞵ-actin* gene was used as an internal control to determine mRNA expression, however, the *U6* gene was used as an internal control for determining miRNA expression [18]. Primers used in real-time PCR were designed and synthesized by ZKYTONG (China) (Table 1). RT-qPCR was carried out in triplicates using the 2× ChamQ SYBR qPCR Master system (Vazyme, Nanjing, China). The reaction was carried out in a LightCycler^®^ real-time fluorescence-based quantitative PCR system (Roche Life Science, Basel, Switzerland). The 2^−∆∆Ct^ method was applied to calculate relative gene expression [19].

### 2.5. Quantitative Assessment of Adipocyte Differentiation Using Lipid (Oil Red O) Staining Assay

Transfected ovine preadipocytes (miR-27a simulant, negative control (NC) simulant (miR-NC), miR-27a inhibitor, NC inhibitor) were induced to differentiate 8 days later. Cells were washed with PBS three times, fixed in 4% formaldehyde formalin for 30 min, and submitted to staining with Oil Red O (Oro) working solution (60% saturated solution and 40% ddH_2_O) [20].

For measuring intracellular lipid content, stained ovine preadipocytes were eluted with pure isopropanol for 1 h, and sample absorbance was measured at 510 nm in an Epoch spectrophotometer (BioTek, Winooski, VT, USA).

### 2.6. Luciferase Reporter Assay

MiR-27a target gene and miRNA binding sites were identified using the Target Scan software (http://www.targetscan.org/vert_72/, accessed on 25 February 2021) [21]. The mature sequence of miR-27a was obtained from the miRBase database. Reporter constructs for luciferase were generated 3′-UTR wild-type (WT) and mutation type (MUT) of *CPT1B* sequences synthesized by ZKYTONG (China) and cloned into the psiCHECK-2 vector (Promega, Madison, WI, USA) at the BamHI sites. Ovine preadipocytes were grown in 12-well plates transfected at 70% confluence using Lipofectamine 3000 reagent (Invitrogen). *CPT1B*-3’UTR WT (0.16 µg) + miR-27a-NC (5 pmol), *CPT1B*-3’UTR WT (0.16 µg) + miR-27a (5 pmol), *CPT1B*-3’UTR MUT (0.16 µg) + miR-27a-NC (5 pmol) and *CPT1B*-3’UTR MUT (0.16 µg) + miR-27a (5 pmol) were transfected into wells containing ovine preadipocytes. Luciferase report assay system (Beyotime, Shanghai, China) was used to evaluate luciferase activity 48 h after transfection.

### 2.7. Western Blot Assay

Ovine preadipocytes were collected using 0.25% trypsin (Solarbio, Beijing, China) and lysed in RIPA buffer (Solarbio) containing 1% PMSF (Pierce, Rockford, IL, USA). Western blot was carried out as described previously [22]. Primary antibodies against β-actin (Abcam 8226) (1:2000, Abcam), *CPT1B* (K004803P) (1:1000, Solarbio), and HRP goat anti-Rabbit IgG secondary antibody (ab97051) (1:2000, Abcam) were used. Chemiluminescent ECL Western blot system (Pierce, Rockford, IL, USA) was used for measuring signal detection.

### 2.8. Data Analysis

Data analysis was performed in GraphPad v.8 (GraphPad Software Inc., San Diego, CA, USA). Data are presented as mean ± SD. Each experiment was performed in triplicate. Student’s t-test was used for pairwise comparisons. Statistical significance was considered at values of *p* < 0.05, *p* < 0.01, and *p* < 0.001.

## 3. Results

### 3.1. Expression of miR-27a and CPT1B under Ovine Preadipocytes Differentiation

**Figure 1:**
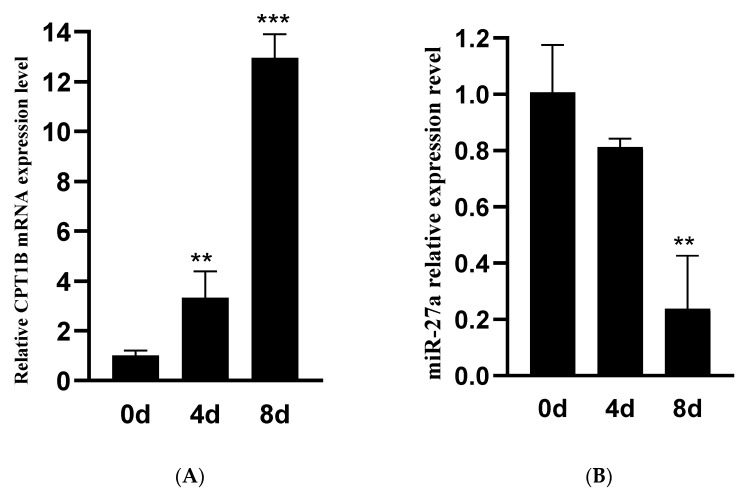
Temporal expression pattern of miR-27a and *CPT1B* under ovine preadipocytes differentiation. Ovine preadipocytes were submitted to induced differentiation for eight days. Cells were collected on days 0, 4, and 8, and expression of *CPT1B* (**A**) and miR-27a (**B**) was determined by RT-PCR. ** p < 0.01, *** p < 0.001.

Ovine preadipocytes were successfully isolated from the LL of Small-tailed Han sheep and submitted to induced differentiation. Temporal patterns of miR-27a and *CPT1B* expression were assessed during ovine preadipocytes adipogenesis. During ovine preadipocytes differentiation, expression of miR-27a gradually decreased (Figure 1A), whereas expression of *CPT1B* gradually increased (Figure 1B). These results suggest that miR-27a displayed a contrary expression pattern with *CPT1B* during ovine preadipocytes differentiation.

### 3.2. MiR-27a Inhibits Differentiation of Ovine Preadipocytes

**Figure 2:**
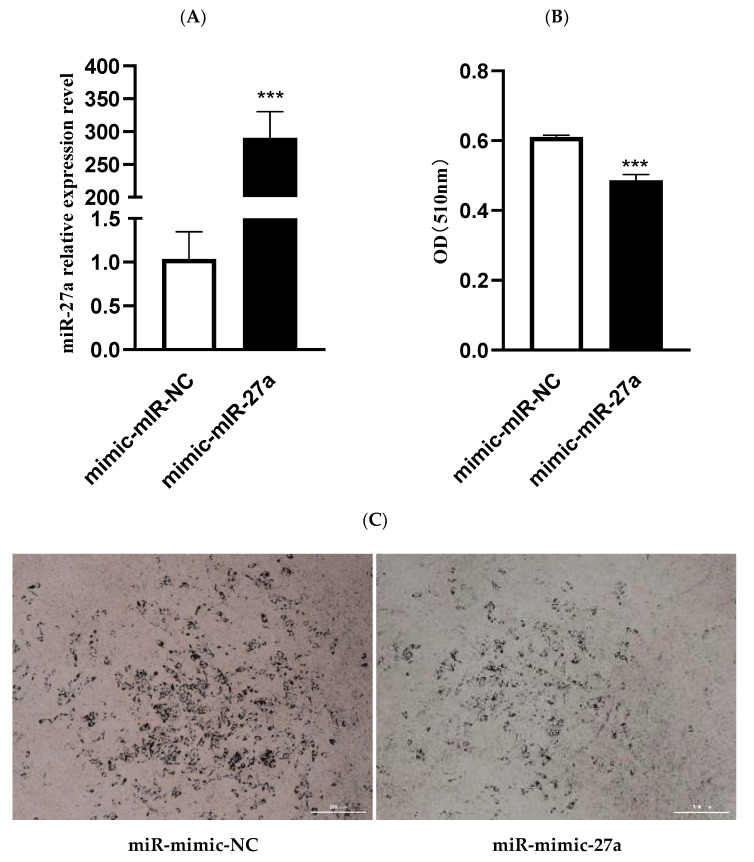
Effect of the miR-27a mimic on ovine preadipocytes. (**A**) miR-27a expression following miR-27a mimic treatment; (**B**) cellular lipid content as determined by spectrophotometry; (**C**) change in lipid droplet accumulation in ovine preadipocytes following miR-27a mimic treatment. *** *p* < 0.001.

**Figure 3:**
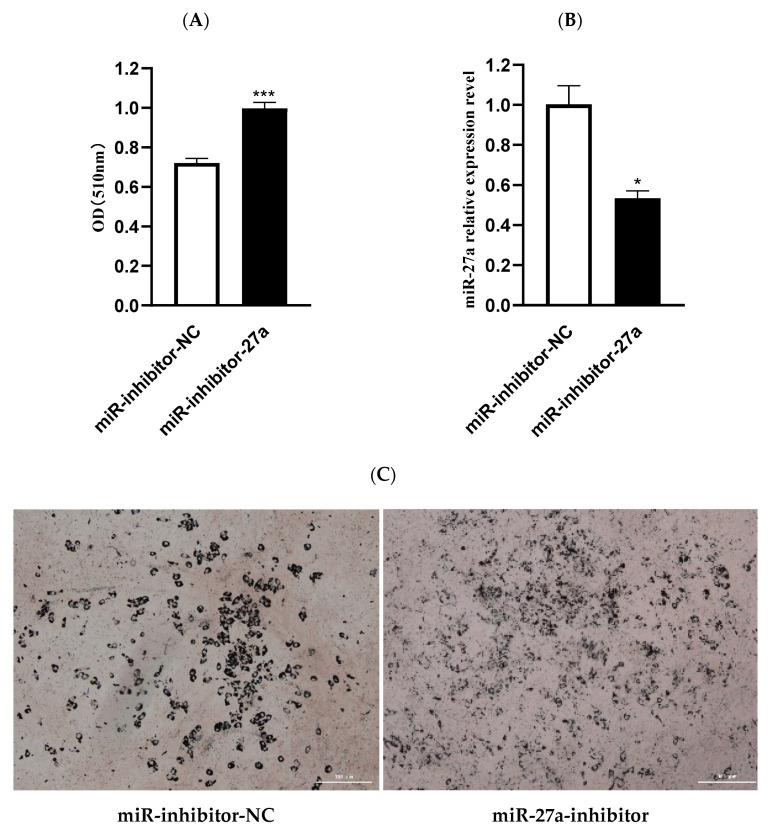
Effect of miR-27a inhibitor on ovine preadipocytes. (**A**) miR-27a expression following miR-27a inhibitor treatment; (**B**) cellular lipid content as determined by spectrophotometry; (**C**) change in lipid droplet accumulation in ovine preadipocytes after treatment with miR-27a inhibitor. * p < 0.05; *** p < 0.001.

The efficiency of miR-27a mimic and inhibitor transfection was confirmed by RT-PCR. miR-27a simulant significantly increased expression of miR-27a (*p* < 0.001) (Figure 2A), while miR-27a inhibitor significantly inhibited expression of miR-27a (*p* < 0.05) (Figure 3A).

To determine the role of miR-27a in lipid metabolism, the absorbance of the sample at 510nm was determined to measure the content of intracellular lipids, and lipid droplet accumulation following overexpression or silencing of miR-27a was determined. miR-27a mimic significantly inhibited the accumulation of lipid droplets compared with the control group (*p* < 0.001) (Figure 2B,C). In contrast, the number of lipid droplets increased significantly when miR-27a was inhibited (*p* < 0.05) (Figure 3B,C). Overall, these findings indicate that miR-27a inhibits the synthesis of lipid droplets in ovine preadipocytes.

### 3.3. MiR-27a Regulates Expression of Genes Related to Lipid Metabolism in Ovine Preadipocytes

**Figure 4:**
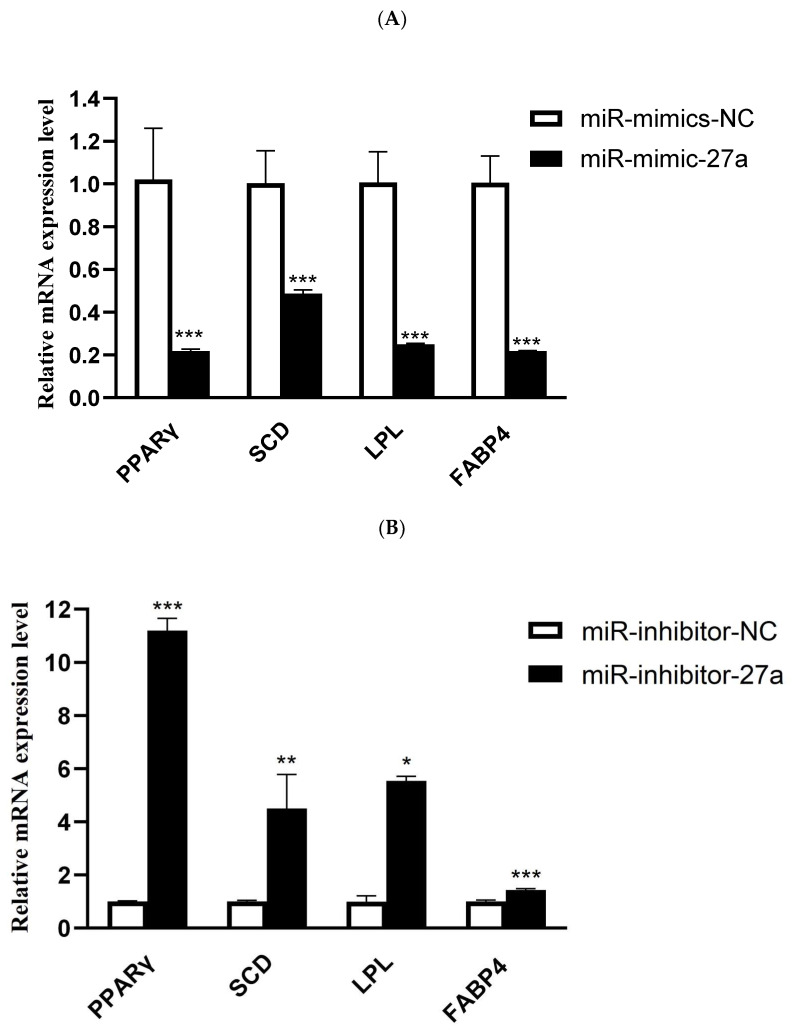
Effect of miR-27a on expression of genes related to lipid metabolism. (**A**) Ovine preadipocytes transfected with miR-27a mimic; (**B**) ovine preadipocytes transfected with miR-27a inhibitor. *** *p* < 0.001.

MiR-27a overexpression significantly reduced expression of genes associated with lipid synthesis, such as *PPAR γ* (*p* < 0.001), *SCD* (*p* < 0.001), *LPL* (*p* < 0.001), and *FABP4* (*p* < 0.001) (Figure 4A). Conversely, transfection of miR-27a inhibitor significantly promoted expression of *PPAR γ* (*p* < 0.001), *SCD* (*p* < 0.01), *LPL* (*p* < 0.05), and *FABP4* (*p* < 0.001) (Figure 4B). Collectively, it can be inferred that miR-27a had a negative effect on the expression of genes related to fat synthesis in ovine preadipocytes.

### 3.4. CPT1B Is a Target Gene for miR-27a

**Figure 5:**
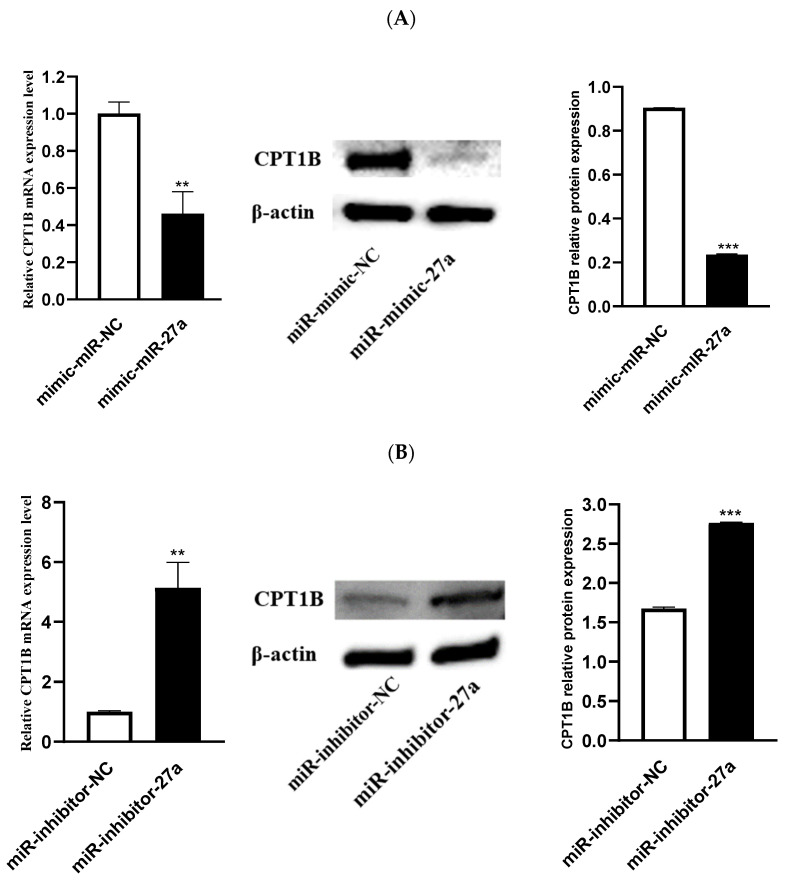
Impact of miR-27a on the *CPT1B* mRNA levels and protein expression. (**A**) Effects of transfection of miR-mimic-NC and miR-mimic-27a on *CPT1B* gene expression and protein in adipocytes. (**B**) Effects of transfection of miR-inhibitor-NC and miR-inhibitor-27a on *CPT1B* gene expression and protein in adipocytes. ** p < 0.01, *** *p* < 0.001.

As shown in Figure 5A, treatment with miR-27a mimic led to downregulation of *CPT1B* expression. In contrast, treatment with miR-27a inhibitor remarkably enhanced *CPT1B* expression (*p* < 0.01) (Figure 5B). Simultaneously, Western blot assay revealed that levels of *CPT1B* protein in miR-27a mimic and inhibitor transfection groups were comparable to those of mRNA expression. Therefore, it can be stated that miR-27a reduced *CPT1B* mRNA levels and protein levels in ovine preadipocytes.

**Figure 6:**
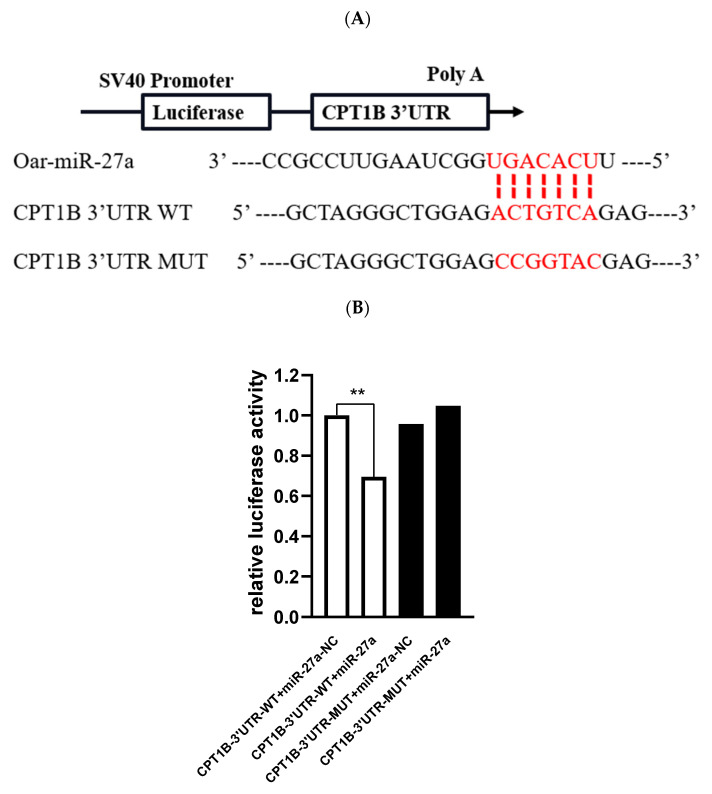
(**A**) Sequence alignment of miR-27a and the 3′-untranslated region (UTR) of *CPT1B* as determined by Target Scan software and miRDB target prediction database; (**B**) changes in luciferase activity after ovine preadipocytes cells were co-transfected with miR-27a mimic and a luciferase reporter with a fragment of the *CPT1B* 3′-UTR harboring either the miR-27a binding site (*CPT1B*-3′UTR-WT) or a mutant (*CPT1B*-3′UTR-MUT). ** p < 0.01. SV40: Simian virus 40; poly A: polyadenylic acid.

To further investigate the mechanism underlying miR-27a inhibition of fat deposition in sheep muscle tissue, we aimed to investigate whether miR-27a targets the 3’-UTR region of *CPT1B*, this region had the binding site for the seed sequence of miR-27a (Figure 6A). Therefore, the luciferase reporter system was used to confirm whether *CPT1B* is a target gene for miR-27a. Co-transfection of miR-27a mimics and *CPT1B-WT-miR-27a* vector directly repressed luciferase activity (Figure 6B; *p* < 0.01), which suggests that *CPT1B* is a direct target gene of miR-27a. Overall, these findings revealed that miR-27a inhibited differentiation and promoted proliferation in ovine preadipocytes by negatively regulating *CPT1B*.

### 3.5. Effect of CPT1B Overexpression on Ovine Preadipocytes Differentiation

**Figure 7:**
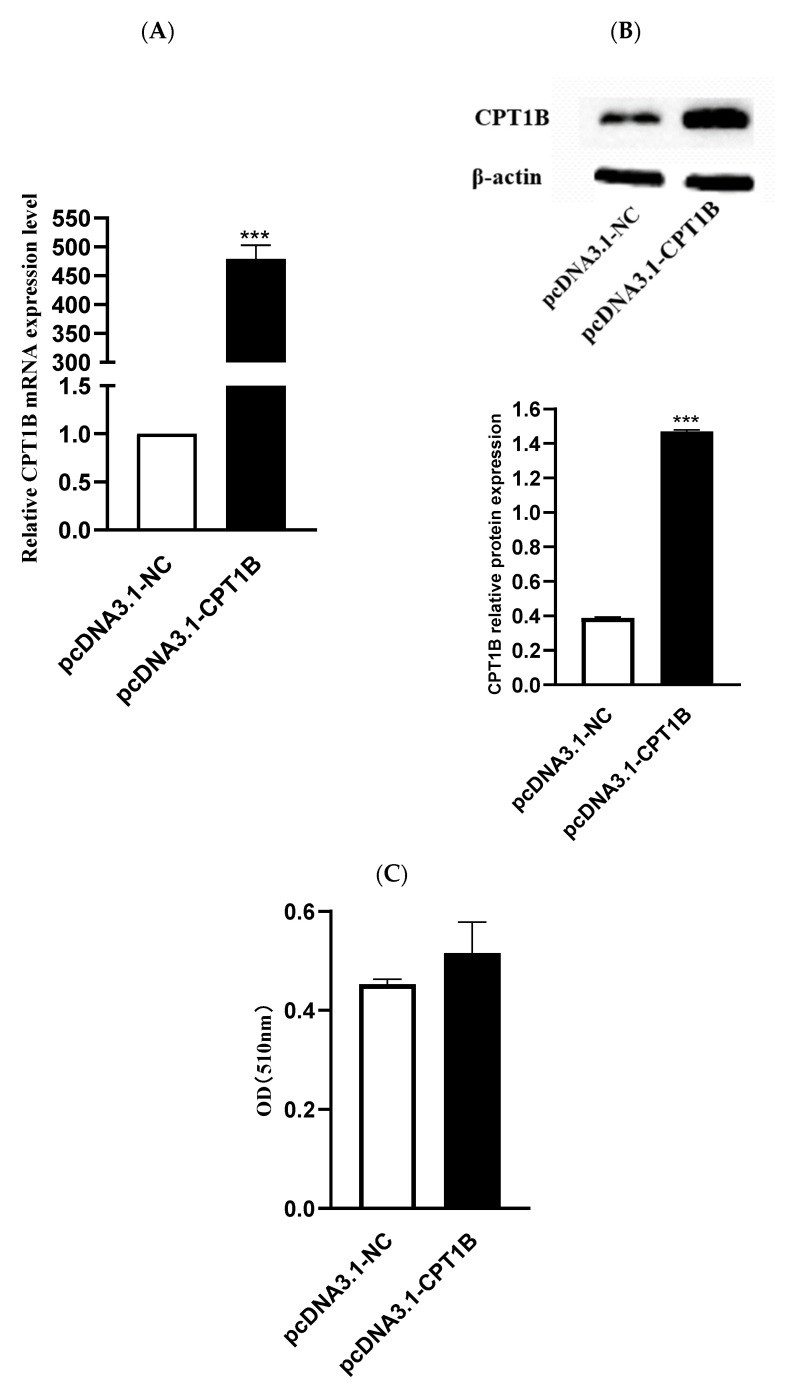
Effect of *CPT1B* on ovine preadipocytes differentiation. (**A**,**B**) Comparative expression of *CPT1B* mRNA and protein levels in ovine preadipocytes transfected with overexpression *CPT1B* vector and *pcDNA3.1-NC* for 48 h; (**C**) cellular lipid content as determined by spectrophotometry. *** p < 0.001.

To reveal the role of *CPT1B* in lipid metabolism of ovine preadipocytes, a *CPT1B* overexpression vector was constructed and transfected into ovine preadipocytes. The optimal transfection concentration was 2.5 µg to yield the highest expression efficiency. Compared with the control group, levels of *CPT1B* mRNA and protein expression in transfected ovine preadipocytes increased significantly (*p* < 0.05) (Figure 7A,B). After 8 days of induction, *CPT1B* overexpression promoted lipid accumulation in ovine preadipocytes (Figure 7C). Collectively, these findings suggest that overexpression of *CPT1B* in ovine preadipocytes promotes adipogenesis.

## 4. Discussion

*CPT1B* is a major isotype of the *CPT* family which comprises other two genes, namely *CPT1A* and *CPT2* [23]. *CPT1B* gene plays a vital role in regulating fat decomposition and energy supply. Studies have shown that *CPT1B* is of great significance to the development of brown adipose tissue [24]. Moreover, in bovine fetal fibroblasts, changes in triglyceride content were related to the expression level of *CPT1B* [25]. It has also been demonstrated that downregulation of *CPT1B* leads to impaired fatty acid oxidation [26]. Furthermore, decreased *CPT1B* protein levels limit the oxidation of cardiac fatty acids [27]. In the present study, overexpression of *CPT1B* led to a significant increase in lipid accumulation in ovine preadipocytes. Interestingly, previous studies have reported that miR-27a inhibits ovine preadipocytes differentiation by regulating the expression of the target gene *RXR* alpha [13]. *CPT1B* is downstream *RXR* alpha in the *PPAR γ* signaling pathway. Therefore, it can be speculated that miR-27a may regulate lipid deposition in ovine preadipocytes via the *PPAR γ* signaling pathway. However, the specific mechanism of regulation needs further elucidation. Based on the above-described results, it can be inferred that miR-27a regulates lipid deposition in ovine preadipocytes by targeting *CPT1B*. Animal fat metabolism is regulated by many factors [28,29,30]. Abnormal fat metabolism leads to increased incidence of a variety of metabolic diseases [31,32]. Therefore, understanding the mechanism underlying the regulation of lipid metabolism has always been the focus of several studies. In recent years, research on miRNA has confirmed that a large number of miRNAs are involved in the regulation of many biological processes related to adipose tissue development [33,34,35]. In previous studies, it has been suggested that *CPT1B* can be a candidate gene for fat deposition in sheep adipose tissue. In addition, it has been hypothesized previously that *CPT1B* may be a potential target gene for miR-27a. In the present study, ovine preadipocytes were employed as a model to explore the regulatory mechanism between miR-27a and *CPT1B* in adipose tissue differentiation, which provided a theoretical basis for further understanding the effect of miRNA on lipid formation in ovine preadipocytes.

The regulatory effect of miR-27a on many types of cells has been previously reported. For instance, silencing miR-27a promoted autophagy and apoptosis of melanoma cells [36], whereas overexpression of miR-27a targeted *GSK-3* *ꞵ* and promoted breast epithelial cell proliferation and invasion [37]. Moreover, miR-27a-3p reduced *ATF3* mRNA and protein levels in a cellular vascular calcification model. [38]. Additionally, miR-27a is closely related to lipid synthesis [39]. MiR-27a affects lipid deposition in IMF and subcutaneous adipose tissue of sheep by participating in adipocyte differentiation and triglyceride synthesis [40,41]. miR-27a and miR-27b target expression of *PPAR γ* in 3T3-L1 cells, thereby controlling the production of lipid droplets [42]. MiR27a can also inhibit *NRF2* expression and contribute to lipid accumulation in hepatocytes [43]. Collectively, the results discussed herein contribute to show that miR-27a negatively regulates lipid production in ovine preadipocytes.

In addition, in the present study, the expression level of miR-27a decreased with differentiation and maturation of ovine preadipocytes, whereas the expression level of *CPT1B* increased with the differentiation and maturation of ovine preadipocytes. These findings are consistent with previous works which showed that the expression level of *the CPT1B* gene increased with an increase in the accumulation of lipid droplets in bovine fetal fibroblasts [25]. The expression trend of miR-27a is opposite to the marker gene *PPAR γ* for adipogenic differentiation [44,45], whereas *PPAR γ* expression level is positively correlated with ovine preadipocytes differentiation. Collectively, these results suggest that miR-27a negatively regulates differentiation of and lipid accumulation in ovine preadipocytes.

Herein, miR-27a was found to inhibit significantly lipid droplet accumulation in ovine preadipocytes, which is consistent with previous studies showing that miR-27a negatively regulates lipid production in ovine preadipocytes [13]. Furthermore, miR-27a was shown to decrease expression of *PPAR γ*, *SCD*, *LPL*, and *FABP4* which are genes related to lipid synthesis in ovine preadipocytes. *PPAR γ* is a positive regulator of lipid accumulation which plays a key regulatory role in adipocyte differentiation [46]. *FABP4* has a significant role in fat deposition and is highly expressed in fat tail [47]. Previous studies showed a possible effect of *SCD* on the regulation of milk fat percentage in Churra sheep [48]. *LPL* plays an important role in regulating fat synthesis in goat breast milk [49]. Our results, therefore, suggest that miR-27a may inhibit the synthesis of lipid droplets by affecting key genes involved in lipid regulation.

## 5. Conclusions

In the present study, miR-27a was shown to be involved in fat synthesis and accumulation in ovine preadipocytes. Specifically, miR-27a regulated the formation of lipid droplets in adipocytes by targeting the *CPT1B* gene. These results may provide a theoretical basis for understanding IMF deposition in sheep muscle tissue.

## Figures and Tables

**Table 1:** The sequence of primers used in the study for mRNA and miRNA quantitative real-time PCR.

Gene Target	Primer Sequence (5′-3′)	Gene ID
*ꞵ-actin*(internal reference of mRNA)	F: ACCGTGAGAAGATGACCCAGA	443052
	R: AGAGGCGTACAGGGACAGCA	
*PPAR γ*	F: TGGATGACCACTCCCATGCC	443513
	R: TTGGGAACGGAATGTCCTC	
*FABP4*	F: GGATGATAAGCTGGTGCTGG	100137067
	R: CTCTGGTAGCAGTGACACCG	
*SCD*	F: TTCATCCTGCCCACACTCG	443185
	R: TAGTTGTGGAAGCCCTCACC	
*LPL*	F: CCCAGCAGCATTATCCAGTGT	443408
	R: ATTCATCCGCCATCCAGTTC	
miR-27a	F: TCGGCAGGTTCACAGTGGCTA	102465824
	R: CTCAACTGGTGTCGTGGAGTC	
*U6* (internal reference of mRNA)	F: CAAGGGCCACATAGATCCG	101121962
	R: AACGCTTCACGAATTTGCGT	
*CPT1B*	F: AGATCCGTATGTTCGACCCAA	443193
	R: CTGCGATCATGTAGGAAACACC	

## Data Availability

The datasets used and/or analyzed during the current study available from the corresponding author on reasonable request.
